# Supplementary material for: kcnj13 regulates pigment cell shapes in zebrafish and has diverged by cis-regulatory evolution between Danio species
Source: Development. 2023 Aug 24;150(16):dev201627. doi: 10.1242/dev.201627 (PMC10482006; doi:10.1242/dev.201627)
Supplement: Supplementary information [file develop-150-201627-s1.pdf]

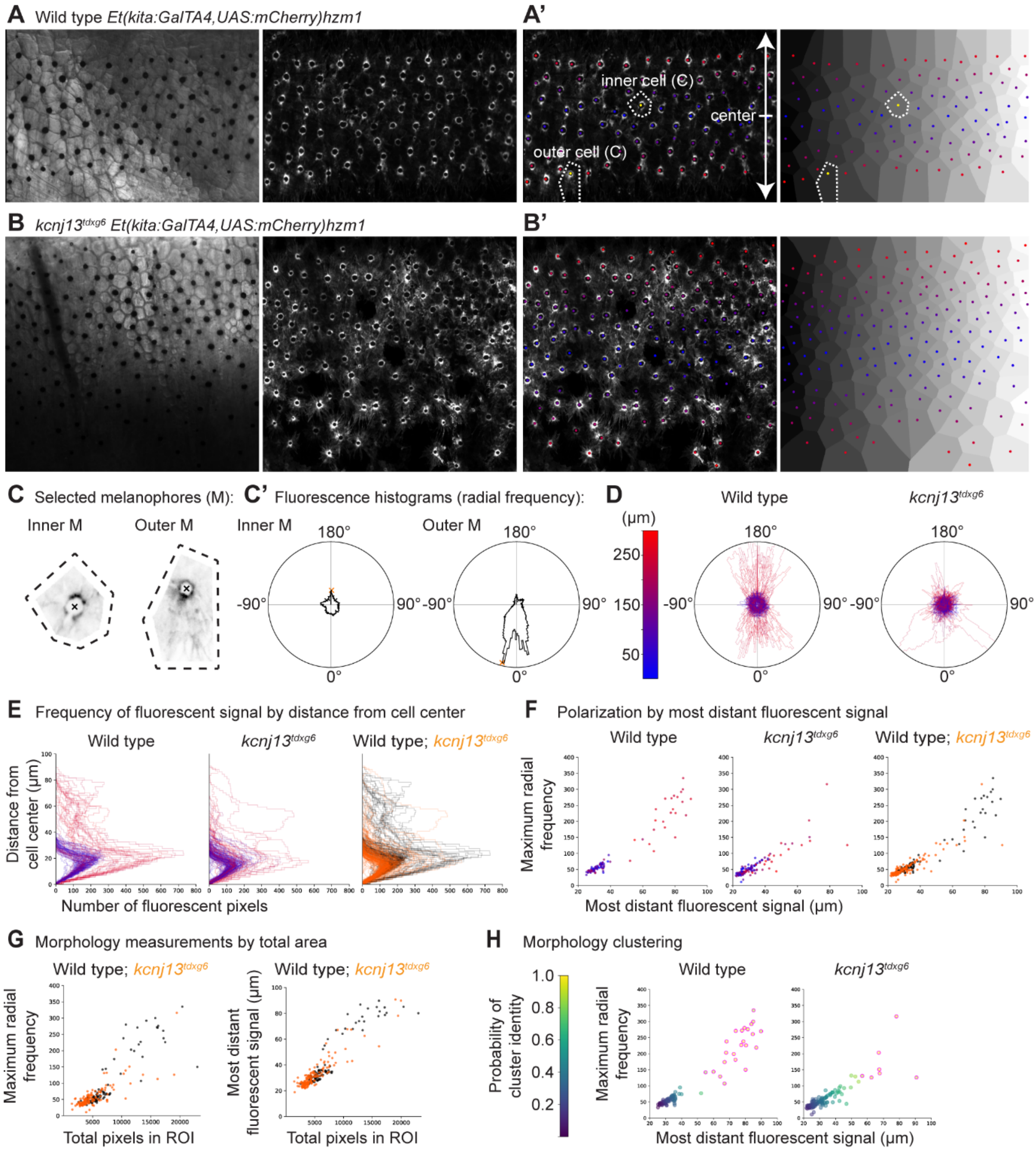

Fig. S1. Quantification of melanophore morphology.

Representative images of A wild-type and B *kcnj13*^{tdxg6} melanophores labelled in *Et(kita:GalTA4,UAS:mCherry)hzm1*, and the corresponding detection of cell centres and automated region of interest segmentation of the images in A’,B’. C Selected inner and outer cells with their radial frequency histogram representations in C’, showing the frequency of fluorescence signal as a function of direction. Orange crosses indicate the maximum radial frequency used in subsequent calculations. D Frequency of fluorescent signal by radial position from the cell centre (polarization)

Development • Supplementary information

for all cells by genotype. E Frequency of fluorescent signal by distance from the cell centre (projection length) for wild-type and mutant cells, indicating wild-type cells have longer projections. F Polarization (maximum radial frequency) plotted by projection length (maximum distance from cell centre) for all cells. G Control plots for both polarization and projection length as a function of the number of pixels in the region of interest, indicating that region size does not account for the measured morphological differences between wild-type and mutant melanophores. H Gaussian Mixture Model clustering of cell morphology for both genotypes, colour coded by the normalized log-likelihood of each cell belonging to the polarized cluster. Cells classified as polarized for subsequent statistical analysis are indicated with magenta outlines. Areas of interest are indicated with dashed lines. Unless otherwise noted, coloration represents cell position within the stripe (reference colourbar).

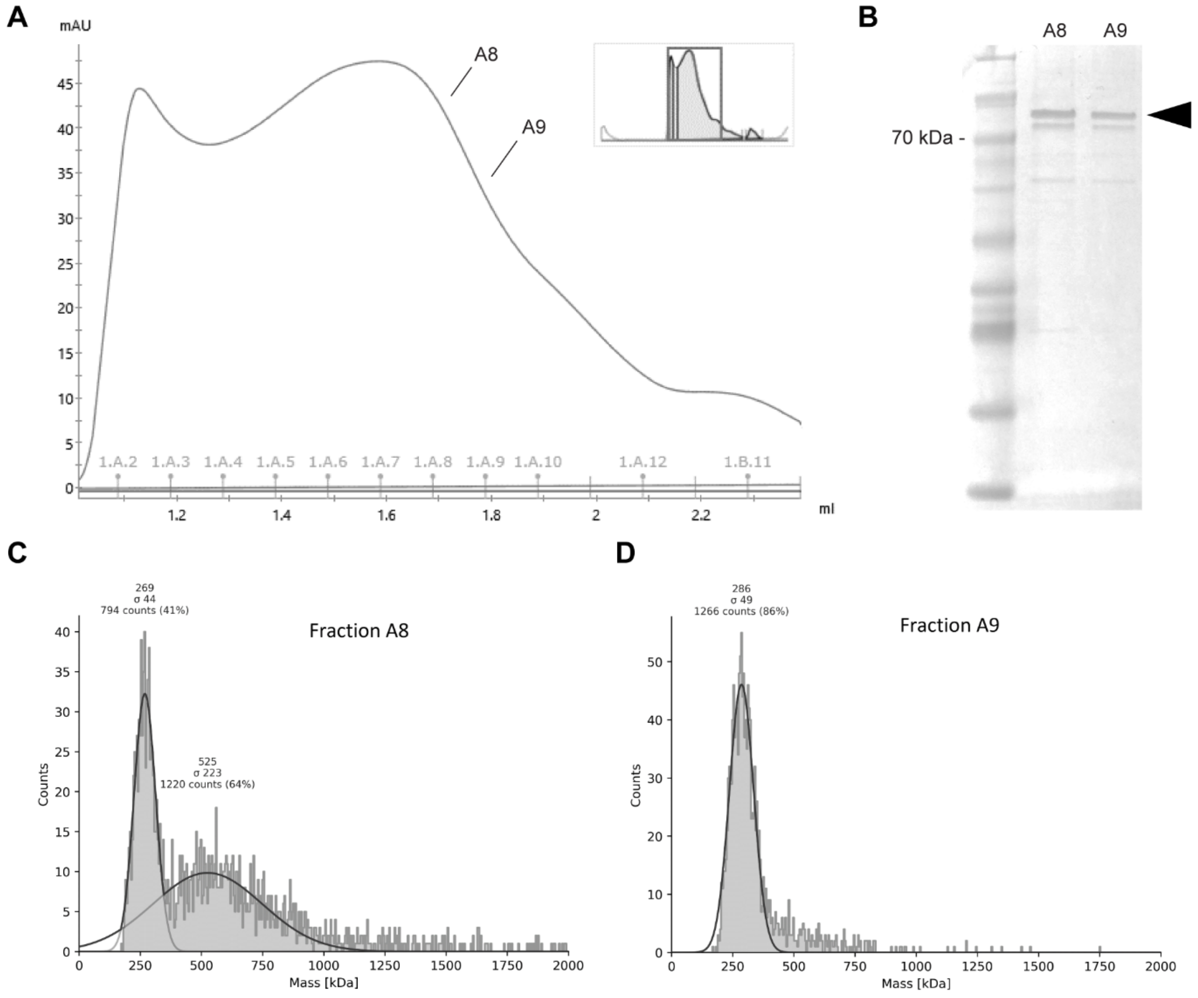

Fig. S2. Protein purification and analysis.

A Size-exclusion chromatogram, fractions A8 and A9 are indicated. B Coomassie staining shows bands corresponding to the expected size of about 70 kDa, with double bands presumably due to glycosylation. C and D show mass-photometry peaks from fractions A8 and A9, corresponding to a molecular mass of about 280 kDa, as expected for a tetrameric complex. In C higher oligomeric states might be present.

Development • Supplementary information

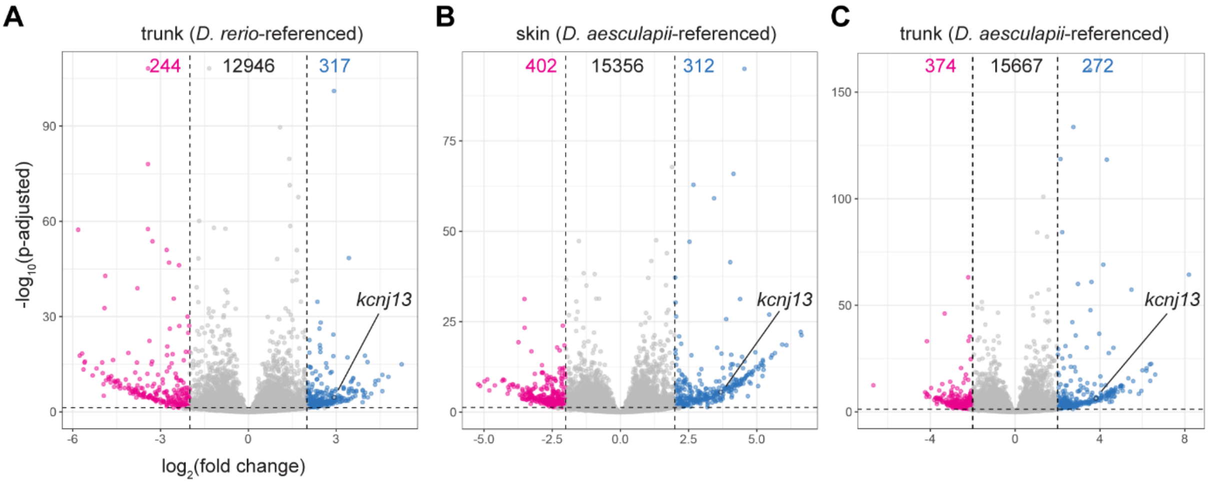

Fig. S3. Allele-specific transcriptome analysis in hybrids between *D. rerio* and *D. aesculapii*.

A Expression analysis in the trunk, using the *D. rerio* reference genome, shows that 317 and 244 genes are significantly higher expressed from either the *D. rerio* (blue) or *D. aesculapii* (magenta) genome, respectively. For most transcripts (12,946) we observed no differences in expression levels. We found significantly higher expression of *kcnj13* from the *D. rerio* allele (*p-adjust* < .00001). B, C Analysis of the same data based on the *D. aesculapii* reference genome yielded very similar results. We found that in the hybrids *kcnj13* was significantly higher expressed from the *D. rerio* genome (p-adjust < .00001 for both B skin and C trunk); although more *D. aesculapii* alleles were identified as higher expressed (402 versus 312, and 374 versus 272 genes in skin or trunk, respectively), possibly due to some mapping bias of the transcripts to the different genome sequences.

Development • Supplementary information

Table S1. New transgenic lines used in this study.

Number	Line
1	*Tg(mitfa:kcnj13$^{D.rerio}$);kcnj13^{t24ui}*
2	*Tg(mitfa:kcnj13$^{D.aesculapii}$);kcnj13^{t24ui}*
3	*Tg(UAS:Venus)*
4	*Tg(kcnj13:KalTA4);Tg(UAS:Venus)*
5	*Tg(kcnj13:KalTA4);Tg(UAS:Venus);slc45a2^{t22mp}*

Table S2. Protein purification buffers.

Buffer	Composition
Lysis buffer A	50 mM HEPES pH 7.5, 100 mM NaCl, 20 mM imidazole, 1 % w/v DDM, 0.5 % w/v CHS, 1 % protease inhibitor (cOmplete Protease Inhibitor Cocktail EDTA-free, Sigma-Aldrich)
Wash buffer B	50 mM HEPES pH 7.5, 100 mM NaCl, 20 mM imidazole, 0.01 % w/v DDM, 0.005 % CHS, 1 % protease inhibitor
Wash buffer C	50 mM HEPES pH 7.5, 100 mM NaCl, 50 mM imidazole, 0.01 % w/v DDM, 0.005 % CHS, 1 % protease inhibitor
Elution buffer D	50 mM HEPES pH 7.5, 100 mM NaCl, 350 mM imidazole, 0.01 % w/v DDM, 0.005 % CHS, 1 % protease inhibitor
Gel filtration buffer E	50 mM HEPES pH 7.5, 100 mM NaCl, 0.01 % w/v DDM, 0.005 % CHS

Development • Supplementary information

Table S3. Transcriptome metadata.

CeGaT ID	Sample description	Sex	Stage	Egg lay date	Sampling date	Extraction date	RIN	Quantity [µg]
S1906Nr1	RNA_skin_rerio-aesculapii_pair1_hybrid_1	NA	adult	20190606	20200113	20190114	9.3	0.81
S1906Nr2	RNA_trunk_rerio-aesculapii_pair1_hybrid_1	NA	adult	20190606	20200113	20190114	9.3	1.75
S1906Nr3	RNA_skin_rerio-aesculapii_pair1_hybrid_2	NA	adult	20190606	20200113	20190114	8.2	1.55
S1906Nr4	RNA_trunk_rerio-aesculapii_pair1_hybrid_2	NA	adult	20190606	20200113	20190114	8.2	1.86
S1906Nr5	RNA_skin_rerio-aesculapii_pair1_hybrid_3	NA	adult	20190606	20200113	20190114	7.9	1.54
S1906Nr6	RNA_trunk_rerio-aesculapii_pair1_hybrid_3	NA	adult	20190606	20200113	20190114	8.2	4.23
S1906Nr7	RNA_skin_rerio-aesculapii_pair1_hybrid_4	NA	adult	20190606	20200113	20190114	8.5	1.43
S1906Nr8	RNA_trunk_rerio-aesculapii_pair1_hybrid_4	NA	adult	20190606	20200113	20190114	9.3	5.70
S1906Nr9	RNA_skin_rerio-aesculapii_pair1_hybrid_5	NA	adult	20190606	20200113	20190114	8.2	1.60
S1906Nr10	RNA_trunk_rerio-aesculapii_pair1_hybrid_5	NA	adult	20190606	20200113	20190114	7.8	3.65
S1906Nr11	RNA_skin_rerio-aesculapii_pair1_hybrid_6	NA	adult	20190606	20200113	20190114	8.5	1.12
S1906Nr12	RNA_trunk_rerio-aesculapii_pair1_hybrid_6	NA	adult	20190606	20200113	20190114	9.2	3.20
S1906Nr13	RNA_skin_rerio-aesculapii_pair1_hybrid_7	NA	adult	20190606	20200113	20190114	9	1.23
S1906Nr14	RNA_trunk_rerio-aesculapii_pair1_hybrid_7	NA	adult	20190606	20200113	20190114	9.6	1.76
S1906Nr15	RNA_skin_rerio-aesculapii_pair2_hybrid_8	NA	adult	20190814	20200113	20190114	9.2	1.56
S1906Nr16	RNA_trunk_rerio-aesculapii_pair2_hybrid_8	NA	adult	20190814	20200113	20190114	9.4	3.77
S1906Nr17	RNA_skin_rerio-aesculapii_pair2_hybrid_9	NA	adult	20190814	20200113	20190114	7.7	1.60
S1906Nr18	RNA_trunk_rerio-aesculapii_pair2_hybrid_9	NA	adult	20190814	20200113	20190114	9.9	2.29
S1906Nr21	RNA_skin_rerio-aesculapii_pair2_hybrid_11	NA	adult	20190814	20200113	20190114	8.8	1.64
S1906Nr22	RNA_trunk_rerio-aesculapii_pair2_hybrid_11	NA	adult	20190814	20200113	20190114	9.7	4.30
S1906Nr25	RNA_skin_rerio-aesculapii_pair2_hybrid_13	NA	adult	20190814	20200113	20190114	9.4	1.37
S1906Nr26	RNA_trunk_rerio-aesculapii_pair2_hybrid_13	NA	adult	20190814	20200113	20190114	10	1.73
S1906Nr27	RNA_skin_rerio-aesculapii_pair2_hybrid_14	NA	adult	20190814	20200113	20190114	9.5	1.51
S1906Nr28	RNA_trunk_rerio-aesculapii_pair2_hybrid_14	NA	adult	20190814	20200113	20190114	9.4	3.41
S1906Nr29	DNA_D_aesculapii_parent3_male_pair1	male	adult	NA	20190612	20190612	NA	2.10
S1906Nr30	DNA_D_rerio_parent1_female_pair1	female	adult	NA	20190612	20190612	NA	5.75
S1906Nr31	DNA_D_rerio_parent5_male_pair2	male	adult	NA	20190816	20190816	NA	8.00
S1906Nr32	DNA_D_aesculapii_parent9_female_pair2	female	adult	NA	20190816	20190816	NA	5.12

Development • Supplementary information
