# Supplementary material for: kcnj13 regulates pigment cell shapes in zebrafish and has diverged by cis-regulatory evolution between Danio species
Source: Development. 2023 Aug 24;150(16):dev201627. doi: 10.1242/dev.201627 (PMC10482006; doi:10.1242/dev.201627)
Supplement: Supplementary file 1 [file develop-150-201627_review_history.pdf]

kcnj13 regulates pigment cell shapes in zebrafish and has diverged by cis-regulatory evolution between *Danio* species

Marco Podobnik, Ajeet P. Singh, Zhenqiang Fu, Christopher M. Dooley, Hans Georg Frohnhoefer, Magdalena Firlej, Sarah J. Stednitz, Hadeer Elhabashy, Simone Weyand, John R. Weir, Jianguo Lu, Christiane Nuesslein-Volhard and Uwe Irion
DOI: 10.1242/dev.201627

Editor: Cassandra Extavour

Review timeline

Original submission:	17 January 2023
Editorial decision:	9 March 2023
First revision received:	22 May 2023
Accepted:	21 July 2023

Original submission

<u>First decision letter</u>

MS ID#: DEVELOP/2023/201627

MS TITLE: *kcnj13* regulates pigment cell shapes in zebrafish and diverged by cis-regulatory evolution between *Danio* species

AUTHORS: Marco Podobnik, Ajeet P. Singh, Zhenqiang Fu, Christopher M. Dooley, Hans Georg Frohnhoefer, Magdalena Firlej, Hadeer Elhabashy, Simone Weyand, John R. Weir, Jianguo Lu, Christiane Nuesslein-Volhard, and Uwe Irion

I have now received all the referees' reports on the above manuscript, and have reached a decision. The referees' comments are appended below, or you can access them online: please go to BenchPress and click on the 'Manuscripts with Decisions' queue in the Author Area.

As you will see, the referees express considerable interest in your work, but have some significant criticisms and recommend a substantial revision of your manuscript before we can consider publication. If you are able to revise the manuscript along the lines suggested, which may involve further experiments, I will be happy receive a revised version of the manuscript. Your revised paper will be re-reviewed by one or more of the original referees, and acceptance of your manuscript will depend on your addressing satisfactorily the reviewers' major concerns. Please also note that Development will normally permit only one round of major revision. If it would be helpful, you are welcome to contact us to discuss your revision in greater detail. Please send us a point-by-point response indicating your plans for addressing the referees' comments, and we will look over this and provide further guidance.

Please attend to all of the reviewers' comments and ensure that you clearly highlight all changes made in the revised manuscript. Please avoid using 'Tracked changes' in Word files as these are lost in PDF conversion. I should be grateful if you would also provide a point-by-point response detailing how you have dealt with the points raised by the reviewers in the 'Response to Reviewers' box. If you do not agree with any of their criticisms or suggestions please explain clearly why this is so.

© 2023. Published by The Company of Biologists under the terms of the Creative Commons Attribution License (https://creativecommons.org/licenses/by/4.0/).

Reviewer 1

Advance summary and potential significance to field

The manuscript of Podobnik et al. describes the role of the kcnj13 gene in the establishment of the pigmented pigmentation stripes during the zebrafish development and its contribution to pigmentation pattern evolution between Danio species. The work relies on a combination of genetics, including new mutant alleles, transgenic lines, a Crispr knock-in, and blastula transplantation experiments to reveal how the potassium channel encoded by kcnj13 is cell-autonomously required in melanophores to instruct the striped pattern.

It was previously shown that kcj13 is necessary for the establishment of the stripe pigmentation pattern in D. rerio. The phenotypic analysis of the kcnj13 mutant shows that this gene is involved in setting the cell shape in melanophore, but also non-autonomously, in the other pigment cell types, and is required to prevent the mixing of the different pigment cell types at the borders of the pigmentation stripes. These two phenomena (the cell shape changes and cell-cell interaction) are presumably functionally linked, however, the exact nature of the connection is not explored in this work.

The knock-in of an optimized Gal4 reporter gene at the kcnj13 locus reveals for the first time the expression pattern of this gene, in particular in the skin at the time of the stripe pattern formation, showing expression in some xanthophores and some melanophores. If we assume that this reporter faithfully captures the full kcnj13 expression pattern then its limited expression to just a few melanophores is quite puzzling given its broad role in the making of the pigmentation stripes, as discussed by the authors.

In parallel to their study of the developmental role of kcnj13 in the making of the pigmentation stripes in D. rerio, the authors also explored further how evolutionary changes at this locus contribute to pigmentation pattern diversification between Dario fishes. The authors previously established with a hemizygosity test that evolutionary changes at the kcnj13 locus contributed to the changes in pigmentation pattern between D. rerio and D. aesculapii, however, the test could not discriminate between changes in the coding sequence (two of them led to an amino acid difference between the species), or in the cis-regulatory sequences, or both. Here, the authors addressed this question first by establishing a kcnj13 mutant rescue experiment in D. rerio using the coding sequence of each of the two species. Each of the two transgenes rescues the D. rerio pattern, suggesting that the 2 amino acid difference does not play any role in the pigmentation pattern divergence. Second, the authors took advantage of species-specific nucleotide sequences to measure the expression of each kcnj13 allele in hybrid fish. In this test the two alleles are in the same hybrid genome, therefore any expression difference between the two kcnj13 alleles must result from functional divergence in the cis-regulatory sequences. The comparison of kcnj13 allelic expression in the skin of the hybrid fish establishes that their expression level differs as a result of divergence in their cis-regulatory sequences. Whether this cis-regulatory divergence leads to spatial expression pattern difference or to a change in expression level remains unknown.

All in all, the authors confirm and extend previous findings about the role of kcnj13 in the making of the pigmentation stripes in D. rerio, and in the diversification of these stripes between closely related fish species. The main merit of the study is to show that kcnj13 is required cell-autonomously in melanophores and controls the shape of, and the interactions with, other pigment cell types. By extension, this gene contributes to setting the boundaries between the different colored striped. The second merit of this study is to show that cis-regulatory divergence at the kcnj13 locus has contributed to shaping the divergent pigmentation patterns between Danio species. The conclusions, which are convincingly supported by the data, improve our understanding of how pigmentation patterns are made and evolve.

Comments for the author

no further comments

© 2023. Published by The Company of Biologists under the terms of the Creative Commons Attribution License (https://creativecommons.org/licenses/by/4.0/).

Reviewer 2

Advance summary and potential significance to field

This is an interesting study focusing on the role of Kcnj13 in pigment pattern formation in Danio species. The authors create kcnj13 knockouts in both D rerio and D aesculapii, showing that pigment pattern is disrupted in each species. Transplants of kcnj13-deficient iridophores (or xanthophores) into an iridophore-deficient background rescues stripe formation in D rerio, showing that kcnj13 is required in melanophores but not xanthophores or iridophores. A kcnj13 reporter line demonstrates patchy expression in different pigment cell types during development, and the authors show that kcnj13 is required to maintain proper cell shapes in chromatophores. Finally, the authors show that the coding region of kcnj13 from D. aesculapii is capable of rescuing pattern formation in a knockout D. rerio, ruling out trans changes as a mechanism for

Comments for the author

Major comments:
In general, statistics throughout would strengthen the paper. As a major conclusion of the paper is that kcnj13 regulates cell shapes, it is necessary to quantify cell shapes in the different conditions. It would also be helpful to measure the length of the melanophore protrusions as long as their polarity in the different conditions.
Additionally, some quantifications of the pigment patterns in different conditions (e.g. measuring the width of the stripes or the width of 'breaks' in stripes) would be beneficial.
Several of the transplantation experiments require controls. Fig 2d should include transplants of WT cells into the slc45ab background. Fig 4d should include sox10:mrfp xanthophores transplanted into a WT background, and shapes of the cells should be quantified in each condition.
Finally, the allele-specific expression of kcnj13 needs to be confirmed by qPCR. If qPCR could be performed both in the hybrid and in each species, this would be much more convincing that trans changes in aesculapii lead to lower kcnj13 expression.

Minor comments:
Both the introduction and the discussion could be greatly condensed. There are several sections which are redundant with one another.
In all the Figures, panels (or legend) should indicate the number of individuals examined. (Additional statistical analysis will also be helpful, see above)
Color brightfield images in especially Fig 3 and also in Fig 4 would be very helpful in telling whether the xanthophores have differentiated and have yellow pigment.

Reviewer 3

Advance summary and potential significance to field

Prior work showed that kcnj13 is important for pigment patterning in Danio rerio but did not test whether this gene was part of a general patterning mechanism or affected only specific pigment cell types. A key and fascinating finding of the current work is that kcnj13 function is required only in melanophores for proper stripe formation. Previous studies suggests that diversity at the kcnj13 locus also may contribute to evolutionary variation in pigment patterns among species of zebrafish. Protein-coding mutant alleles caused patterning defects but whether coding or regulatory differences contributed to variation between species was unknown. Here, the authors make a strong case for cis-regulatory divergence between species. Overall, I found this to be an exciting study that will generate interest among developmental biologists interested in zebrafish, pigmentation, and evolution.

Comments for the author

Line 308. An allele-specific expression assay is a great experiment to test for cis-regulatory divergence between alleles. However, certain challenges arise in RNA-seq experiments that involve

© 2023. Published by The Company of Biologists under the terms of the Creative Commons Attribution License (https://creativecommons.org/licenses/by/4.0/).

mapping transcripts of two species onto the reference genome of only of the species. There is a risk that the transcripts of the reference species will be a better match to the reference genome, potentially resulting in an accurate count of the reference species transcripts (D. rerio in this case) and an undercount of transcripts from the non-reference species (D. aesculapii). I couldn't tell from the Methods section if this potential bias was taken into account in this study. For both tissues tested, D. rerio kcnj13 transcript abundance was higher, which is the expected result of a bias due to using the D. rerio reference genome.

Could the authors please clarify if they took this potential pitfall into account in their analysis? One way around this problem is to do quantitative RT-PCR targeting a highly-conserved part of the transcript. I wouldn't ordinarily suggest this step because it is usually redundant to RNA-seq data, but it could help clarify the results in a case like this with potentially divergent transcript sequences. For example, the authors of a paper conducting a broadly similar allele-specific expression test on inter-species hybrids found conflicting expression results from their RNA-seq and RT-PCR experiments, presumably due to transcript sequence divergence between species (Lopes et al, 2016, Current Biology 26: 1427-1434). If the authors of the current manuscript have already corrected for this issue in some way, they should make this clearer in the Methods section or elsewhere.

L 441. The authors establish that the function of the protein is probably similar between species, yet the shape of WT D. aesculapii melanophores is similar to those of D. rerio with mutant alleles. Can the authors speculate about why might this be? How might expression level of kcnj13 affect cell morphology?

L 496. The Hart et al. paper that identified trans-acting factors noted that cis-regulatory changes also contributed, but the cis-acting changes varied among populations.

L 510. Supplementary Fig. 2 shows differential expression of kcnj13 in the trunk, so the cis-regulatory change is not limited to skin expression. The magnitude of difference in the trunk is lower than the magnitude in the skin, but the differential expression analysis still flags the difference as significant. If this difference persists after addressing my first comment above, please change the text to reflect this broader result, which contradicts the conclusion of "presumably non-pleiotropic" regulatory evolution.

First revision

Author response to reviewers' comments

Point by point response to the reviewers' comments:

Reviewer 2:

In general, statistics throughout would strengthen the paper. As a major conclusion of the paper is that kcnj13 regulates cell shapes, it is necessary to quantify cell shapes in the different conditions. It would also be helpful to measure the length of the melanophore protrusions as long as their polarity in the different conditions.

We agree with the reviewer that a more quantitative and statistical analysis could strengthen our conclusions regarding differences in cell morphology. To address this, we applied an unsupervised automated image analysis method to measure both the polarity and the projection length of melanophores within the stripes of wild-type and mutant fish (refer to new Methods section lines 748-764). This approach identified more polarized cells in the stripe margins of wild-type fish. Further, we conducted a statistical comparison of these data between genotypes (refer to text changes lines 238-245). We now include a supplemental figure showing the additional requested analyses (Fig. S1).

© 2023. Published by The Company of Biologists under the terms of the Creative Commons Attribution License (https://creativecommons.org/licenses/by/4.0/).

Additionally, some quantifications of the pigment patterns in different conditions (e.g. measuring the width of the stripes or the width of 'breaks' in stripes) would be beneficial.

We also agree that pattern quantifications could be beneficial; however, there are several reasons why we did not pursue this:
- The patterns in the mutants are obviously different from wild type, there is little risk of mis-categorization.
- There is, however, considerable variability in the mutant patterns, which is, at least partly, due to the genetic background of the different fish lines, as we show in the paper.
- Therefore, a thorough quantification taking a whole range of different parameters into account would be necessary to try and capture more subtle differences in the phenotypes. This is beyond the scope of the current manuscript and will be addressed in the future.

Several of the transplantation experiments require controls. Fig 2d should include transplants of WT cells into the slc45ab background. Fig 4d should include sox10:mrfp xanthophores transplanted into a WT background, and shapes of the cells should be quantified in each condition.

The 'controls' for these transplantation experiments, i.e. transplants of wild-type cells into the corresponding donors were published previously. We put the corresponding references into the figure legends.

Finally, the allele-specific expression of kcnj13 needs to be confirmed by qPCR. If qPCR could be performed both in the hybrid and in each species, this would be much more convincing that trans changes in aesculapii lead to lower kcnj13 expression.

Due to the very high similarity of the *kcnj13* transcripts from the different species designing oligonucleotide primers for qPCR experiments proved very challenging. Therefore, to rule out a mapping bias in our analysis (see comment from Reviewer 3), we re-analysed the RNA-seq data using the *Danio aesculapii* genome as reference. This analysis confirmed the higher expression of *kcnj13* in the hybrids from the *D. rerio* genome in both, skin and trunk samples.

Both the introduction and the discussion could be greatly condensed. There are several sections which are redundant with one another.

We tried to be as concise as possible, while still making the manuscript accessible for non-expert readers.

In all the Figures, panels (or legend) should indicate the number of individuals examined. (Additional statistical analysis will also be helpful, see above)

We have added those numbers to the figure legends.

Color brightfield images in especially Fig 3 and also in Fig 4 would be very helpful in telling whether the xanthophores have differentiated and have yellow pigment.

Unfortunately colour bright-field images of the different panels from Fig. 3 and Fig. 4 were not taken at the time of imaging.

Reviewer 3:

Line 308. An allele-specific expression assay is a great experiment to test for cis-regulatory divergence between alleles. However, certain challenges arise in RNA-seq experiments that involve mapping transcripts of two species onto the reference genome of only of the species. There is a risk that the transcripts of the reference species will be a better match to the reference genome, potentially resulting in an accurate count of the reference species transcripts (D. rerio in this case) and an undercount of transcripts from the non-reference species (D.

© 2023. Published by The Company of Biologists under the terms of the Creative Commons Attribution License (https://creativecommons.org/licenses/by/4.0/).

aesculapii). I couldn't tell from the Methods section if this potential bias was taken into account in this study. For both tissues tested, D. rerio kcnj13 transcript abundance was higher, which is the expected result of a bias due to using the D. rerio reference genome. Could the authors please clarify if they took this potential pitfall into account in their analysis? One way around this problem is to do quantitative RT-PCR targeting a highly-conserved part of the transcript. I wouldn't ordinarily suggest this step because it is usually redundant to RNA-seq data, but it could help clarify the results in a case like this with potentially divergent transcript sequences. For example, the authors of a paper conducting a broadly similar allele-specific expression test on inter-species hybrids found conflicting expression results from their RNA-seq and RT-PCR experiments, presumably due to transcript sequence divergence between species (Lopes et al, 2016, Current Biology 26: 1427-1434). If the authors of the current manuscript have already corrected for this issue in some way, they should make this clearer in the Methods section or elsewhere.

A potential bias in favour of the transcripts derived from the reference species could indeed be a major concern for the RNA-seq analysis in hybrids. Initially we also considered qPCR to rule this out. However, we found that the *kcnj13* transcripts from both species are highly similar, which makes it very difficult to design primer pairs that work well for qPCR and simultaneously distinguish between the two alleles. At the same time, this high similarity makes a bias less likely. With the availability of a reference genome for the second species, *Danio aesculapii*, it was possible to re-analyse the data. For *kcnj13* we still find significantly higher expression from the *D. rerio* allele when using the *D. aesculapii* genome as reference. This shows that our assumption that the high similarity of the transcripts would allow both alleles to be mapped to the genome equally well was probably correct.

L 441. The authors establish that the function of the protein is probably similar between species, yet the shape of WT D. aesculapii melanophores is similar to those of D. rerio with mutant alleles. Can the authors speculate about why might this be? How might expression level of kcnj13 affect cell morphology?

Unfortunately we do not have a clear understanding of a mechanism how *kcnj13* affects cell shapes. So far, it would be mere speculation to assume any direct influence of the (probably) altered membrane potential of a cell on its shape. Equally well possible would be an indirect effect on the differentiation state of the cells, which might result in shape changes. We feel that statements in any of these directions would be too speculative for now.

L 496. The Hart et al. paper that identified trans-acting factors noted that cis-regulatory changes also contributed, but the cis-acting changes varied among populations.

We changed this in the text.

L 510. Supplementary Fig. 2 shows differential expression of kcnj13 in the trunk, so the cis-regulatory change is not limited to skin expression. The magnitude of difference in the trunk is lower than the magnitude in the skin, but the differential expression analysis still flags the difference as significant. If this difference persists after addressing my first comment above, please change the text to reflect this broader result, which contradicts the conclusion of "presumably non-pleiotropic" regulatory evolution.

We clarified the text to make it more obvious that the skin is also part of the trunk in our experimental set up. Therefore, we would expect that differences are still present. We apologize for not stating this more clearly in the original version of the manuscript.

© 2023. Published by The Company of Biologists under the terms of the Creative Commons Attribution License (https://creativecommons.org/licenses/by/4.0/).

Second decision letter

MS ID#: DEVELOP/2023/201627

MS TITLE: *kcnj13* regulates pigment cell shapes in zebrafish and diverged by cis-regulatory evolution between *Danio* species

AUTHORS: Marco Podobnik, Ajeet P. Singh, Zhenqiang Fu, Christopher M. Dooley, Hans Georg Frohnhoefer, Magdalena Firlej, Sarah J. Stednitz, Hadeer Elhabashy, Simone Weyand, John R. Weir, Jianguo Lu, Christiane Nuesslein-Volhard, and Uwe Irion
ARTICLE TYPE: Research Article

First, please accept my sincere apologies for the unacceptable length of time it has taken for me to gather reviews, make a decision, and relay it to you. This was due to a combination of later reviewer response and to my own schedule, but it is not an acceptable time frame for authors like yourself who submit their work to Development, and for that I apologise.

I am happy to tell you that your manuscript has been accepted for publication in Development, pending our standard ethics checks.

Reviewer 1

Advance summary and potential significance to field

The main merit of the study is to show that kcnj13 is required cell-autonomously in melanophores and controls the shape of, and the interactions with, other pigment cell types. By extension, this gene contributes to setting the boundaries between the different colored striped. The second merit of this study is to show that cis-regulatory divergence at the kcnj13 locus has contributed to shaping the divergent pigmentation patterns between Danio species. The conclusions, which are convincingly supported by the data, improve our understanding of how pigmentation patterns are made and evolve.

Comments for the author

I think the authors have properly addressed all the concerns raised by the reviewers.

Reviewer 2

Advance summary and potential significance to field

This is an interesting study focusing on the role of Kcnj13 in pigment pattern formation in Danio species. The authors create kcnj13 knockouts in both D rerio and D aesculapii, showing that pigment pattern is disrupted in each species. Transplants of kcnj13-deficient iridophores (or xanthophores) into an iridophore-deficient background rescues stripe formation in D rerio, showing that kcnj13 is required in melanophores but not xanthophores or iridophores. A kcnj13 reporter line demonstrates patchy expression in different pigment cell types during development, and the authors show that kcnj13 is required to maintain proper cell shapes in chromatophores. Finally, the authors show that the coding region of kcnj13 from D. aesculapii is capable of rescuing pattern formation in a knockout D. rerio.

Comments for the author

Fig. 1 is missing panel labels.
The authors cite previous works transplanting WT cells into mutant hosts, however the cell shapes have not been quantified in any of the conditions. Could the methods used in Supp Fig 1 be applied in other analyses as well?

© 2023. Published by The Company of Biologists under the terms of the Creative Commons Attribution License (https://creativecommons.org/licenses/by/4.0/).

It is disappointing that the experiment recommended by both reviewers (qPCR analysis of kcnj13 in hybrids) is not feasible.

Reviewer 3

Advance summary and potential significance to field

Prior work showed that kcnj13 is important for pigment patterning in Danio rerio but did not test whether this gene was part of a general patterning mechanism or affected only specific pigment cell types. A key and fascinating finding of the current work is that kcnj13 function is required only in melanophores for proper stripe formation. Previous studies suggests that diversity at the kcnj13 locus also may contribute to evolutionary variation in pigment patterns among species of zebrafish. Protein-coding mutant alleles caused patterning defects but whether coding or regulatory differences contributed to variation between species was unknown. Here, the authors make a strong case for cis-regulatory divergence between species. Overall, I found this to be an exciting study that will generate interest among developmental biologists interested in zebrafish, pigmentation, and evolution.

Comments for the author

I am happy with the authors' responses to my comments, particularly the mapping of transcripts to the other species' reference genome. I look forward to the publication of this paper.

© 2023. Published by The Company of Biologists under the terms of the Creative Commons Attribution License (https://creativecommons.org/licenses/by/4.0/).
